# Climate change: impact on livestock and how can we adapt

**DOI:** 10.1093/af/vfy039

**Published:** 2019-01-03

**Authors:** Umberto Bernabucci

**Affiliations:** 1 Department of Agriculture and Forests Science, University of Tuscia-Viterbo, Italy; 2 Department of Excellence, Ministry for Education, University and Research of Italy (Law 232/216)


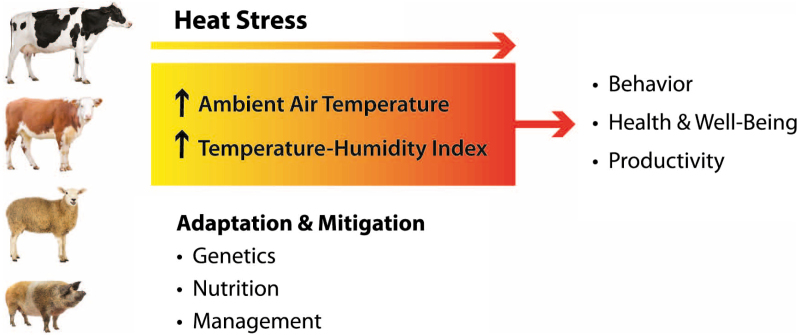


This issue of Animal Frontiers, “Climate change: impact on livestock and how can we adapt,” focuses on the effects of climate change (global warming) on livestock health, well-being, production and reproduction, and on possible adaptation and mitigation strategies that can be put in place to reduce negative impacts.

Recently the intergovernmental group of experts on climate change gathered in South Korea to bring attention to the urgency of this situation: global warming is increasing and ecosystems, animal species diversity, and food security are at risk. It is now well accepted that the increasing concern with the thermal comfort of agricultural animals is justifiable not only for countries in tropical zones, but also for nations in temperate zones where high-ambient temperatures are becoming an issue. At a global level, animal production must increase in the next decades to satisfy the growing need for animal-sourced foods. We have to expect that livestock systems (based on grazing, mixed farming systems, or industrialized systems) will be more and more negatively affected by climate change, especially global warming.

The article by Pasqui and Di Giuseppe (2019) clearly shows that climate is changing. In addition to the increase in temperature, there is an increase in the frequency of extreme events such as the number of hot days and the number of heat waves. Heat waves are the combination of duration and intensity of air temperature and can strongly affect human activities as well as the health and productivity of farm animals. In recent decades, the scientific community generated much new knowledge of the fundamental mechanisms of the Earth’s climate system as well as the implications and impacts of climate change. Contemporarily, an effort has been directed to identify new actions for mitigating the anthropogenic greenhouse gas emission trends, and on identifying new actions to adapt to the observed and expected changes in climate.

In the last quarter century, the livestock sector was focused on improving productivity, modifying the environment, and improving nutritional management rather than improving stress resistance. This approach dramatically increased productivity of domestic animals but also increased their sensitivity (reduced their thermal plasticity) to hot environments. The processes by which domestic animals respond to changes in their environment are critical to survival but often negatively affect productivity and profitability of livestock systems. Understanding how these processes are controlled will offer opportunities for improving thermal stress resistance. Collier et al. (2019) describe the meaning of acclimation, acclimatization, and adaptation to environmental stressors, with emphasis on heat stress. Acclimation and acclimatization are a coordinated phenotypic response to environmental stressors and the response will decay if the stressors are removed. If chronic stress persists over several generations, the acclimatization response will become genetically “fixed” and the animal will be adapted to the environment. Improving knowledge of the genetic differences between adapted animals will contribute useful information of the genes associated with acclimation. This information will be useful to help identify genes associated with improved thermotolerance.

Rust (2019) clearly shows how climate change affects both extensive and intensive livestock production systems, with emphasis on adaptation. Improving knowledge of the impact of climate change on different livestock systems and the adaptation strategies to fight climate change are of vital importance. Livestock systems, especially in developing countries, are extremely dynamic and the size and relative production output, especially in intensive animal farming practices, are increasing around the world to satisfy the growing demand for livestock products, especially in some areas characterized by adverse climatic conditions. Extensive and intensive livestock production systems will be affected differently by climate change and, thus, different adaptation strategies must be implemented.

Heat stress undoubtedly negatively affects animal health and welfare. Lacetera (2019) outlines how a hot environment affects farm animal health and further describes the direct and indirect effects of heat stress. The direct effects are due primarily to increased temperatures and frequency and intensity of heat waves. These environmental conditions can affect livestock health by causing metabolic disruptions, oxidative stress, and immune suppression causing infections and death. The indirect effects are those linked to alteration of the availability and the quality of feedstuffs and drinking water as well as survival and redistribution of pathogens and/or their vectors. Development and application of new methods, tools, and techniques to link climate data with disease surveillance systems should be implemented in the future for improving prevention of diseases as well as improved mitigation and adaptation responses of animals to heat stress.

Wolfenson and Roth (2019) describe how hot summer conditions disrupt several reproductive processes, resulting in a pronounced depression of conception rate in dairy cows worldwide. When body temperature reaches 39.5 °C a strong impairment of reproductive processes such as disruption of oocyte developmental competence, attenuated embryonic growth and early embryonic death due to impairment of hormone secretion, alteration of ovarian follicular growth dynamics, suboptimal development of the corpus luteum, and attenuated uterine endometrial responses may occur. Application of efficient cooling is a must and a prerequisite to minimize heat stress. However, sometimes it is not enough to lessen heat stress during summer to sustain reproductive function even when the stressor ends. It is suggested that cooling must be combined with other treatments to improve fertility. In particular, treatments for improving the timing of ovulation, enhanced removal of impaired follicles, induction of ovulation of healthy follicles, embryo transfer, and progesterone supplementation before and after artificial insemination may be needed to improve fertility of heat-stressed dairy cows.

Heat stress negatively affects milk and meat production. In addition to quantity, the quality of animal products is strongly and negatively affected by a hot environment. With regard to milk, heat stress has a more important effect on high-quality products such as the protected designation of origin cheeses from many European countries that have a world-renown reputation for excellence. Summer et al. (2019) point out the negative effects of heat stress on the composition of milk (organic and inorganic components) and describe how those changes are strongly associated with the alteration of cheesemaking properties and the merchandise value of milk. These changes result in significant, negative economic impacts for producers and consumers. Beef cattle, with their lower metabolic rate and lower body heat production, are usually considered less sensitive to heat stress than dairy cattle. However, beef cattle also compensate for increased body temperature by homeostatic mechanisms (panting, sweating, and urination) and behavioral alterations such as reduced activity, increased water intake, and reduced feed intake. These effects are responsible for generally lower growth rate and reduced fertility of both males and females.

Gaughan et al. (2019) address an important topic that is and will be a source of debate among researchers: why animals have to adapt and which strategies will be the best for adaptation? Animal adaptation is a function of several factors which are interrelated. All factors that will either enhance or reduce adaptability must be considered. It is well known that selection of animals for high levels of production has increased animal susceptibility to environmental challenges. On the other hand, using lower production cows could reduce heat stress, but reduced production efficiency may lead to increased greenhouse gas intensity. Even if a single stressor may be important, the cumulative effects of multiple stressors (in addition to heat stress) may be significant and must be considered. Adaptation strategies include production system adjustments and genetic improvement for thermotolerance. In addition to adaptation, mitigation strategies should also be addressed. These include changes in animal management systems (nutritional interventions, manipulation of the rumen eco-system, provision of shade, housing, fans, and sprinklers). Multidisciplinary approaches including animal breeding, nutrition, housing, and health are required for reducing the adverse impact of climate change on livestock.

Globally, pork is one of the most consumed animal-sourced foods. Reduced and inconsistent growth, decreased feed efficiency, decreased carcass quality (increased lipid deposition and decreased protein accretion), poor sow performance, decreased reproductive performance (male and female), increased mortality (especially in sows and market hogs), and morbidity are the main economic losses associated with heat stress in the swine industry (Mayorga et al., 2019). Evidence suggests that maternal exposure to heat stress has long-lasting effects on postnatal offspring performance. The combination of climate change forecasts, increased pork production in tropical and subtropical regions of the globe and improved genetic capacity for lean tissue accretion and fecundity, all point to increasingly negative impacts of heat stress on pork production efficiency and quality in the future. Physically modifying the environment is currently the primary abatement strategy that should be utilized to mitigate the negative effects of heat stress. Additional approaches including dietary modifications and genetic improvement may help improve mitigation and adaptation of pigs to adverse environmental conditions.

The article by Carabaño et al. (2019) explores the possibility of selecting farm animals for thermotolerance. Genetic selection is a cost-effective tool to achieve a permanent change in an animal’s tolerance to heat, even though implementing selection strategies is challenging because of the complexity of the heat stress response and the antagonism between heat tolerance and productivity. To effectively select animals, there is a need to find phenotypic measures that accurately identify heat-tolerant animals and that can be used under field conditions with low cost. In addition, developing methods to efficiently combine knowledge from all “omics” technologies to produce genetic indices to perform selection of the best breeding stock is needed. Genetic improvement for heat-tolerant livestock is effective according to the production system. Systems that can provide enough resources to insure high productivity of animals will benefit more from including heat tolerance in the breeding programs of the already selected breeds for high production. In contrast, production systems with scarce resources and harsh parasite environments will benefit more from crossing local stock with highly specialized, productive breeds.

Grossi et al. (2019) debate a different topic from those addressed in the previous articles. This article focuses on the effects of livestock on the climate and discusses the main greenhouse gas emissions of the livestock sector. The livestock sector requires a significant amount of natural resources and is responsible for greenhouse gas emissions (methane and nitrous oxide). Greenhouse gases mainly come from enteric fermentation, manure storage and feed production. Implementation of mitigation strategies aimed at reducing emissions from the livestock sector is needed to limit the environmental burden from food production while ensuring a sufficient supply of food for a growing world population. Mitigation may occur directly by reducing the amount of greenhouse gases emitted or indirectly through the improvement of production efficiency. To increase the effectiveness of these strategies, the complex interactions among the components of livestock production systems must be taken into account to avoid environmental trade-offs.

Food and water security will be one of the priorities for human kind in the future. During this same time, the world will experience a change in the global climate that will cause shifts in the local climate that will affect local and global agriculture. It is now accepted that warming of the climate is unequivocal and anthropogenic warming will continue due to time scales associated with climate processes and feedback. Surface air warming in the 21st century, by best estimates, will range from 1.1 to 2.9 °C for a “low scenario” and 2.4 to 6.4 °C for a “high scenario.” Decision makers, research institutions, and extension services must support livestock activities to cope at best with the loss of production efficiency, decreased quality of animal products, and enlargement of land desertiﬁcation and the worsening of animal health under the expected effects of climate change in the next decades.

## About the Author

**Umberto Bernabucci** is a professor in Environmental Physiology and Ruminant Nutrition at the Department of Agriculture and Forests Science, University of Tuscia-Viterbo, Italy. He is active, for over 20 yr, in the areas of feeding and management of heat-stressed dairy cows. He is Chair of the Master Courses in Agricultural Biotechnology and Director of the Clinical Biochemistry and Immunology Lab at the Department of Agriculture and Forests Science. His current research involves studies on the following: interactions between lipid metabolism, metabolic diseases, immune response, and oxidative status in dairy ruminants with particular attention to the peripartum period; adipokines and energy homeostasis; effects of heat stress on physiology, health, and performance of dairy cows; and nutraceutical properties of milk and cheeses.
